# Evaluation of Serum Complement Components in Pediatric IgA Vasculitis: A Case-Control Study

**DOI:** 10.3390/children12081090

**Published:** 2025-08-20

**Authors:** Raziye Burcu Taşkın, Güzide Aksu, Sait Şen, Gülden Hakverdi, Burçe Emine Dörtkardeşler, Secil Conkar Tunçay

**Affiliations:** 1 Department of Pediatric Rheumatology, Faculty of Medicine, Health Sciences University Tepecik Training and Research Hospital, Konak, Izmir 35020, Turkey; raziyeburcu.taskin@saglik.gov.tr; 2 Department of Pediatric Rheumatology and Immunology, Faculty of Medicine, Ege University, Bornova, Izmir 35100, Turkey; guzide.aksu@ege.edu.tr; 3 Department of Pathology, Faculty of Medicine, Ege University, Bornova, Izmir 35100, Turkey; sait.sen.ege@edu.tr; 4 Department of Biostatistics, Faculty of Medicine, Cumhuriyet University, Sivas 58140, Turkey; ghakverdi@cumhuriyet.edu.tr; 5 Department of Pediatrics, Faculty of Medicine, Ege University, Bornova, Izmir 35100, Turkey; burce.dortkardesler@ege.edu.tr; 6 Department of Pediatric Nephrology, Faculty of Medicine, Ege University, Bornova, Izmir 35100, Turkey

**Keywords:** IgA vasculitis, nephritis, complement factor H, pediatrics

## Abstract

**Highlights:**

**What are the main findings?**

- Pediatric IgAV patients showed significantly reduced serum CFH levels compared to controls, regardless of nephritis status.
- Serum levels of sC5b-9, MBL, and MASP-1 did not differ between patients and healthy children.

**What is the implication of the main finding?**

- Reduced CFH levels suggest a role of alternative complement pathway dysregulation in IgAV pathogenesis.
- sC5b-9, MBL, and MASP-1 are unlikely to serve as noninvasive biomarkers for renal involvement in IgAV.

**Abstract:**

Background: IgA vasculitis (IgAV) represents the most frequently seen form of vasculitis among children. Although it often resolves without intervention, renal involvement (IgAV nephritis) poses a risk for long-term complications. Although the lectin and alternative complement pathways are possible causes in its development, dependable serum biomarkers for the early identification of nephritis remain unavailable. Methods: In this prospective case-control study, we examined how the serum levels of a membrane attack complex (sC5b-9), complement factor H (CFH), mannose-binding lectin (MBL), and mannose-binding lectin-associated serine protease-1 (MASP-1) relate to renal involvement in IgAV. These complement proteins were measured in children diagnosed with IgAV and compared to levels in healthy controls (HCs) matched for age and sex. Results: The study cohort comprised 44 IgAV patients with a median age of 8 years and 34 HCs. The CFH levels were reduced significantly in the patient group (median: 357.31 ng/mL; IQR: 228.32) relative to the controls (median: 543.08 ng/mL; IQR: 504.05) (p < 0.001). This decrease was observed irrespective of the presence of nephritis. There were no significant differences in serum sC5b-9, MBL, or MASP-1 levels between the patients and controls. Furthermore, no correlation emerged between these complement components and renal involvement. Conclusion: The data suggest that lower CFH levels may signal systemic dysregulation of the alternative pathway in IgAV. In contrast, the serum levels of sC5b-9, MBL, and MASP-1 appear inadequate as markers for predicting renal involvement. Further research with larger cohorts that includes genetic analyses and examination of kidney tissue is needed to better define the contribution of complement activation in IgAV-related nephritis.

## 1. Introduction

Immunoglobulin A vasculitis (IgAV), previously termed Henoch–Schönlein purpura, is the most common pediatric vasculitis and may present with isolated skin involvement or with multi-organ involvement [1,2]. When systemic, IgAV most commonly affects the joints, gastrointestinal tract, and kidneys. Most children with IgAV experience spontaneous resolution, with an overall favorable prognosis observed in approximately 95% of cases. However, a minority (1–2%) of the patients develop end-stage kidney disease (ESKD), highlighting the importance of nephritis severity in determining long-term outcomes [3]. Consequently, early identification and close monitoring of renal involvement are essential for optimal patient management.

Studies on complement activation in IgA nephropathy (IgAN), a disease sharing immunopathogenic features with IgAV, have paved the way for understanding its role in IgAV. Previous studies have shown IgA, C3, and properdin collection in the mesangium and skin biopsies of patients with IgAN and IgAV nephritis (IgAVN), which suggests that an alternative complement pathway plays a role in both conditions [4,5]. Later tests showed mesangial deposits of mannan-binding lectin (MBL) and MBL-associated serine protease 1 (MASP-1), along with high plasma levels of C4d, suggesting that the lectin pathway had been activated [6,7,8]. Additionally, mesangial deposits containing terminal complement complexes, including the membrane attack complex (MAC; C5b-9), have been identified in both IgAN and IgAVN [9].

Together, these results suggest that the alternative, lectin, and terminal complement pathways play a role in both IgAN and IgAVN. The lectin pathway begins when MBL, ficolins, or collectins trigger MASP-1, which then breaks down C4 and C2. On the other hand, the alternative pathway is always slightly active and helps boost the complement response. Complement factor H (CFH) keeps this pathway under control by reducing the activity of C3 convertase and preventing overactivation through negative feedback. These pathways converge at C3 activation, ultimately generating the anaphylatoxins C3a and C5a—potent mediators of inflammation—and the terminal C5b-9 complex, which induces cell lysis [10].

Nevertheless, much of what is known about complement activation in IgAVN is derived from histological studies reporting glomerular complement deposition [4,5,6,7,8,9]. Meanwhile, studies on IgAN have investigated urinary complement products as potential non-invasive biomarkers [11,12]. Although increasing evidence has demonstrated complement component presence in blood, skin lesions, and kidney biopsy specimens from patients with IgAV, no serum complement protein has yet been validated as a reliable biomarker to identify kidney involvement at an early stage in IgAV or IgAVN [13,14,15]. This gap highlights the importance of investigating serum alternative and lectin complement component levels in these patients.

Against this backdrop, our study aimed to investigate whether circulating complement proteins linked to the alternative, lectin, and terminal pathways—including soluble C5b-9 (sC5b-9), MBL, MASP-1, and CFH—could help identify renal involvement in children with IgAV.

## 2. Materials and Methods

### 2.1. Study Design and Participants

From April 2021 to January 2024, a prospective case-control study was carried out at the Pediatric Nephrology and Rheumatology Departments of Ege University Faculty of Medicine to examine whether sC5b-9, CFH, MBL, and MASP-1 are linked to a higher risk of developing IgAV. Therefore, a cohort of age- and sex-matched healthy controls (HCs) was recruited for comparison. IgAV diagnosis was established according to the 2008 Ankara classification criteria [16]. The inclusion criteria also required disease onset between 2 and 18 years of age, at least 6 months of follow-up, and a new diagnosis established at our center. Patients were excluded if they had platelet counts below 100,000/mm^3^, no active purpura, a history of immunosuppressive therapy, other inflammatory or renal diseases, follow-up shorter than six months, or if consent was not provided. The HCs were recruited from children seen for routine check-ups at the Department of Child Health and Diseases, Ege University Faculty of Medicine, who had no systemic or dermatological conditions.

To further assess the potential relationship between the selected complement proteins and nephritis status or severity, the patients were divided into subgroups. Firstly, serum complement protein levels were compared between patients with IgAVN and those without nephritis (IgAVwN). IgAVN was characterized by albuminuria (albumin-to-creatinine ratio exceeding 30 mg/mmol), microscopic hematuria (≥5 red blood cells per high-power field), or an estimated glomerular filtration rate (eGFR) below 60 mL/min/1.73 m^2^. Subsequently, patients with nephritis were stratified into subgroups according to the nephritis severity (mild, moderate, severe, or persistent proteinuria), based on the SHARE (Single Hub and Access point for pediatric Rheumatology in Europe) recommendations [17]. These subgroups were compared with each other and with the IgAVwN and HC groups regarding the selected complement components.

### 2.2. Data Collection

At diagnosis and prior to treatment, demographic data (age, sex), clinical presentation, and blood samples for baseline laboratory parameters were collected. The baseline laboratory investigations included complete blood count, serum creatinine, urea, IgA, serum complement components C3 and C4, antistreptolysin O (ASO), acute phase reactants such as serum amyloid A (SAA) and C-reactive protein (CRP), urinalysis, spot urine protein-to-creatinine ratio, and fecal occult blood testing.

For the complement analysis, in addition to baseline blood samples obtained at diagnosis, one more blood sample was collected from each patient and HC in an EDTA-containing tube. The collected samples were stored at −80 °C until analysis. The levels of sC5b-9, CFH, MBL, and MASP-1 were measured using ELISA kits (Bt-laboratory, China): Human MAC ELISA Kit, Human MBL ELISA Kit, MASP-1 ELISA Kit, and Human CFH ELISA Kit. All measurements were performed in duplicate.

Renal biopsy was indicated for patients with persistent hematuria, proteinuria (protein-to-creatinine ratio > 0.5 g/g), or impaired renal function. Two independent pathologists, blinded to the clinical data, evaluated the biopsy specimens. Immunofluorescence confirmed mesangial IgA deposits. Histopathological features were classified using the updated Oxford MEST-C criteria as follows:

- Mesangial hypercellularity: M0 (≤50% glomeruli), M1 (>50%);
- Endocapillary hypercellularity: E0 (absent), E1 (present);
- Segmental glomerulosclerosis: S0 (absent), S1 (present);
- Tubular atrophy/interstitial fibrosis: T0 (0–25%), T1 (26–50%), T2 (>50%);
- Crescents: C0 (none), C1 (<25% glomeruli), C2 (≥25%) [18].

Data from follow-up visits were collected prospectively for six months using standardized forms, with assessments performed by a pediatric nephrologist or rheumatologist.

### 2.3. Statistical Analysis

All statistical evaluations were carried out using IBM SPSS Statistics (version 25.0) and GraphPad Prism (version 10.0). The distribution of continuous variables was tested for normality using the Shapiro–Wilk test when the sample size was below 50 and the Kolmogorov–Smirnov test for larger datasets. Variables following a normal distribution are expressed as mean ± standard deviation, while non-normally distributed data are reported as median values along with their ranges (minimum–maximum) or interquartile range (IQR). To compare two independent groups, the independent samples *t*-test was used for the normally distributed variables, and the Mann–Whitney U test was applied for those not meeting the normality assumption. For analyses involving more than two groups, the Kruskal–Wallis test was utilized, with Dunn’s multiple comparisons post hoc test and Bonferroni adjustment applied when needed. The categorical data are summarized as frequencies and percentages. The group differences for the categorical variables were assessed using Pearson’s chi-square test or Fisher’s exact test, depending on the expected cell counts. A p-value of less than 0.05 was regarded as statistically significant.

### 2.4. Ethical Statement

Parental or legal guardian written consent was secured prior to participation. The study was approved by the Ethics Committee of Ege University Faculty of Medicine (decision no: 21-7T/32, 8 July 2021) and was managed under the principles of the Helsinki Declaration.

## 3. Results

A sum of 44 pediatric patients with IgAV (20 males, 24 females; median age of 8 and 34 age- and sex-matched HCs) were enrolled in the study. There were no significant differences regarding age or sex distribution between the groups. All patients presented with palpable purpura. Gastrointestinal involvement occurred in 18 cases (40.9%), and joint involvement was noted in 10 children (22.7%). Renal involvement was identified in 18 patients (40.9%), with 11 undergoing renal biopsy. All patients with nephritis had hematuria, and proteinuria was found in 11 (25%). Serum creatinine concentrations were normal across all subjects. The median time from the onset of symptoms to the diagnosis of nephritis was 20 days (range: 12.5–82.5). Biopsies were performed at a median of 18.5 days (range: 10–50) after urinary abnormalities were first detected. The data are shown in Table 1.

**Figure 1:**
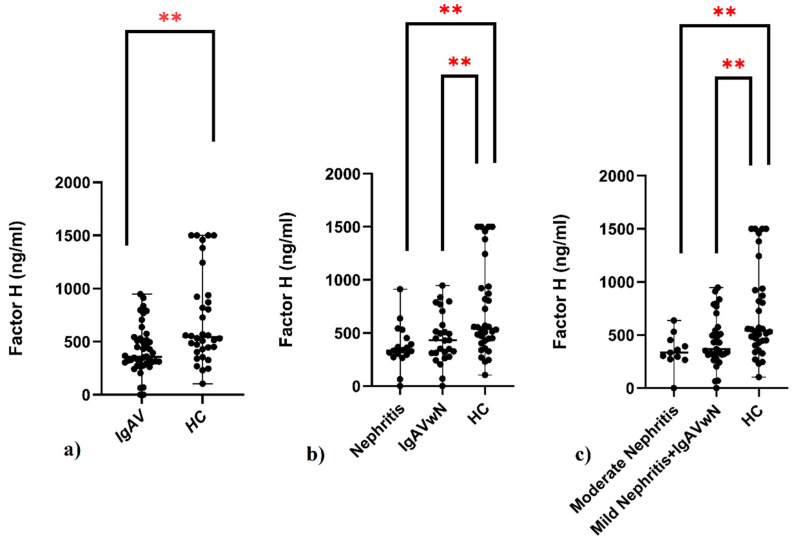
Serum complement factor H (ng/mL) in children with IgAV and HCs. (**a**) Comparison of factor H levels between IgAV patients and HCs. (**b**) Comparison of factor H levels among three groups: patients with nephritis, IgAVwN, and HCs. (**c**) Comparison of factor H levels between nephritis severity subgroups (mild and moderate nephritis), IgAVwN, and HCs. IgAV: immunoglobulin A vasculitis; IgAVwN: IgAV without nephritis; HCs: healthy controls. Data are expressed as medians and analyzed using the Mann–Whitney U test and Kruskal–Wallis test with Dunn’s multiple comparison test. ** p < 0.05.

**Figure 2:**
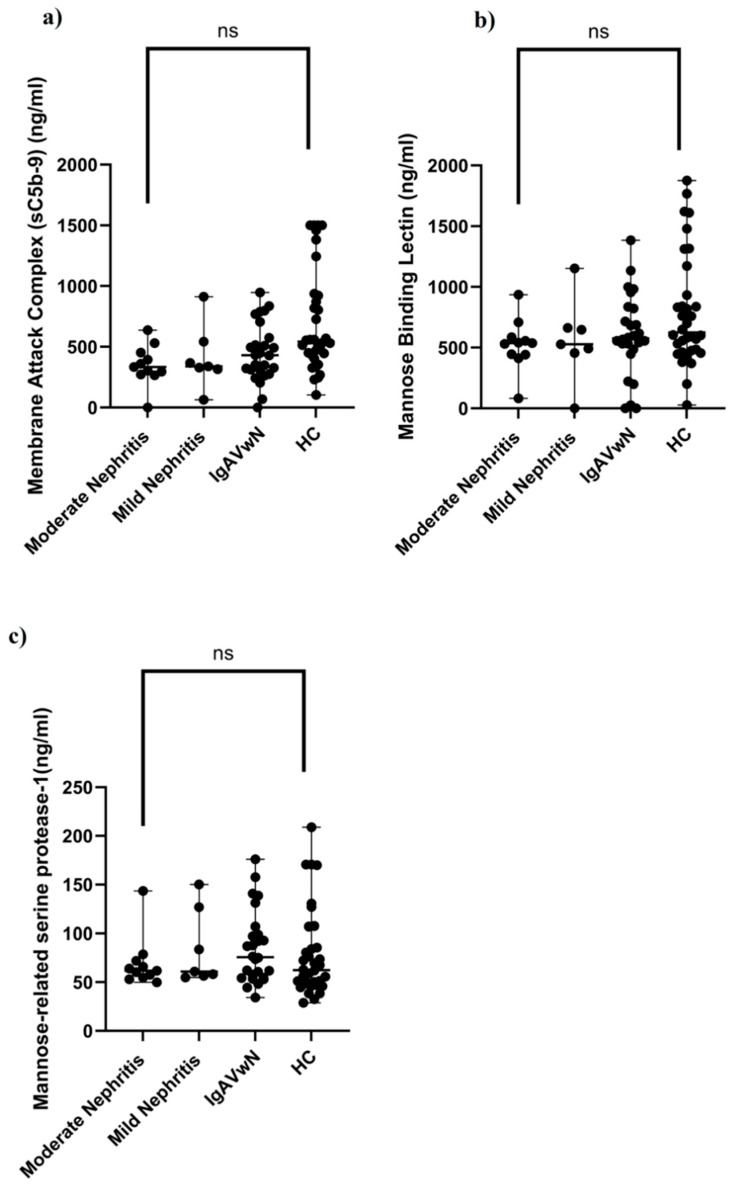
Serum complement concentrations in patients with IgAVwN, mild nephritis, moderate nephritis, and HCs. (**a**) Membrane attack complex (sC5b-9) (ng/mL). (**b**) Mannose-binding lectin (ng/mL). (**c**) Mannose-related serine protease-1 (ng/mL). IgAVwN: immunoglobulin A vasculitis without nephritis; HCs: healthy controls; ns: not significant. Data are expressed as medians and analyzed using the Kruskal–Wallis test with Dunn’s multiple comparison test. ns: p > 0.05.

In order to assess the potential involvement of the alternative and lectin complement pathways in IgAV and renal manifestations, serum concentrations of sC5b-9, CFH, MBL, and MASP-1 were measured in both patients and controls. The IgAV group showed markedly lower CFH concentrations (median: 357.31 ng/mL, IQR: 228.32) compared to the HC group (median: 543.08 ng/mL, IQR: 504.05), with the difference being statistically significant (p < 0.001) (Table 1, Figure 1a). Both the nephritis subgroup (median: 336.53 ng/mL, IQR: 181.32) and the non-nephritis subgroup (median: 430.21 ng/mL, IQR: 306.60) had significantly reduced CFH levels in comparison to the controls (p < 0.05 for both) (Figure 1b). Within the nephritis group, CFH concentrations were the lowest in those classified as having moderate nephritis (median: 334.56 ng/mL, IQR: 178.86), a level significantly below that of the control group (p < 0.05) (Table 2; Figure 1c). However, serum levels of sC5b-9, MBL, and MASP-1 did not significantly differ among IgAV patients with and without nephritis, between the mild and moderate nephritis subgroups, or when any of these groups were compared to HCs (Table 1 and Table 2; Figure 2).

There were no significant differences in serum IgA, C3, C4, ASO, SAA, and CRP levels among patients with IgAVwN, mild nephritis, or moderate nephritis (all p > 0.05). The median (IQR) values for each group were as follows, respectively: C3: 140.5 (24), 141 (9), and 138 (15) mg/dL (p = 0.81); C4: 27.5 (10), 22 (15), and 24 (12) mg/dL (p = 0.72); ASO: 222 (264), 210 (262), and 148 (259) IU/mL (p = 0.92); IgA: 145 (68.25), 146 (69.12), and 140 (40) g/L (p = 0.71); SAA: 45 (101.25), 67 (87), and 78 (90) mg/L (p = 0.64); and CRP: 11 (16.25), 15 (5), and 11 (11) mg/L (p = 0.34). The biopsy results and corresponding complement marker levels for the 11 biopsied patients are presented in Table 3; a statistical analysis was not conducted because of the limited sample size.

## 4. Discussion

Herein, we evaluated the role of alternative, lectin, and terminal pathway complement components in the pathogenesis of IgAV and its renal complications. Our results revealed that serum CFH—a critical regulator of the alternative pathway—was significantly decreased in children with IgAV compared to HCs. Notably, CFH levels were similarly reduced in patients with and without nephritis, suggesting that alternative pathway dysregulation represents a systemic characteristic of IgAV rather than being confined to renal involvement. This is the first investigation to evaluate these particular complement markers in a Turkish pediatric population with IgAV.

Low levels or functional changes in complement regulators like CFH—often due to genetic variants—have been linked to autoimmune and inflammatory diseases such as IgAN, systemic lupus erythematosus, and several kidney disorders [19,20,21]. The current understanding is that factor H-related (FHR) proteins can antagonize the regulatory function of CFH on complement activation [22]. A recent study on rheumatoid arthritis showed that CFH was mostly missing in the synovial lining but was found in large amounts in the tissue beneath it, making the area more prone to activation of the alternative complement pathway. This local deficiency of CFH, combined with the presence of FHR proteins, likely promotes complement-mediated synovial injury and significantly lowers CFH expression, which is correlated with higher disease activity and radiographic joint damage, underscoring its protective role [23].

Decreased CFH concentrations have similarly been reported in IgAN, further supporting its potential role in disease pathogenesis [24]. CFH helps control complement activation both in the bloodstream and on cell surfaces [25]. Therefore, reduced CFH levels in the blood may reflect increased activity of the alternative pathway. In our study, all patient groups had lower CFH levels than the HCs, suggesting that weakened complement regulation may play a role in the development of IgAV. This reduction may reflect increased CFH consumption due to ongoing complement activation or could result from genetic variants affecting CFH synthesis or function. Although a previous study has suggested that specific CFH polymorphisms may protect against severe renal involvement in IgAV by maintaining higher CFH levels [26], the absence of a significant difference between patients with and without nephritis in our cohort indicates that CFH alone may not serve as a reliable biomarker for predicting renal involvement. Instead, our findings suggest a more generalized systemic complement imbalance in IgAV, highlighting the potential pathogenic relevance of low CFH levels.

Regarding the lectin pathway, previous studies have investigated the roles of MBL and MASP-1 in IgAV and IgAN, but the findings on their serum levels have been inconsistent. One earlier study with a small group of IgAVN patients showed that MBL and MASP-1 were present in the glomeruli. However, serum MBL levels did not differ significantly between IgAVN patients and control groups, and no clear link was found between glomerular deposits and clinical outcomes [7]. In contrast, a recently conducted study revealed meaningfully elevated plasma MASP-1 levels in IgAV patients compared to HCs, although these elevations did not correlate with proteinuria or histopathological findings [27].

Furthermore, a genetic polymorphism study identified an MBL2 variant associated with absent MBL expression in both serum and renal tissue, which was strongly linked to a higher risk of ESRD in IgAN [28]. Another study demonstrated a nonlinear association between serum MBL concentrations and clinical outcomes in IgAN, with both low and high MBL levels associated with adverse features such as recurrent infections, gross hematuria, and proteinuria [29]. Collectively, these findings suggest that systemic measurements of lectin pathway components may not reliably capture localized complement activation or renal involvement in IgAV. In line with the hypothesis that the clinical utility of serum MBL and MASP-1 levels may be limited, we did not find significant differences in these markers between IgAV patients and HCs. Taken together, the current body of evidence underscores the need for a more comprehensive approach to evaluating lectin pathway activation in IgAV, incorporating tissue-level analysis, urinary biomarkers, and genetic profiling rather than relying solely on serum concentrations.

Lastly, as for the terminal complement system, our study revealed no significant differences regarding serum levels of sC5b-9 between IgAV patients (with or without nephritis) and HCs. These findings are parallel to the findings of Wijaya et al. [30], who reported no association between plasma sC5b-9 levels and glomerular terminal complement complex (TCC) deposits. This supports the concept that systemic complement activation markers and local complement deposition in tissues may reflect separate pathological processes. Whereas TCC deposition in the kidney is a marker of sustained, localized complement activation, circulating sC5b-9 levels typically indicate systemic or acute complement activity. The absence of elevated sC5b-9 in our cohort may reflect transient or already-resolved systemic activation at the time of sampling rather than an absence of complement involvement altogether. Nonetheless, as renal tissue complement deposits were not evaluated in this study, we did not have the chance to assess the extent of local complement activation in nephritis cases.

In summary, our findings suggest that serum levels of sC5b-9, MBL, and MASP-1 are unlikely to serve as reliable biomarkers for identifying or evaluating renal involvement in IgAV, nor do they appear to indicate an inherent predisposition to the disease. The absence of kidney tissue complement analysis in this study limits our ability to fully understand local complement activity. Future research should focus on combining serum measurements with renal tissue studies in larger, biopsy-confirmed cohorts to better elucidate the role of these complement components in IgAV.

This study is subject to certain limitations that should be noted. The relatively small sample size—especially within the nephritis severity subgroups—may have limited our ability to detect more subtle or nuanced associations. Complement protein levels were measured at a single time point, which prevented us from evaluating changes over time or during different stages of the disease. Although kidney biopsies were assessed according to the Oxford MEST-C classification, we did not examine complement deposition within renal tissue, which limited our understanding of local complement activity. Additionally, potential genetic factors that might affect CFH production or function were not investigated.

Despite this study’s limitations, our results add to the accumulating evidence highlighting the role of the alternative complement pathway in IgAV and suggest that decreased CFH levels may represent a meaningful disease-related marker.

## 5. Conclusions

Our results suggest that alternative pathway dysregulation, reflected by lower serum CFH levels, may be responsible for the development of IgAV in children. In contrast, systemic levels of sC5b-9, MBL, and MASP-1 showed no variation across patient subgroups, limiting their utility as biomarkers for detecting renal involvement. Future studies involving larger, well-characterized cohorts that combine genetic analysis and renal tissue complement evaluation are needed to clarify the predictive value of CFH and further define the role of complement activation in IgAV nephritis.

## Figures and Tables

**Table 1:** Demographics, clinical characteristics, and serum complement levels of the subjects.

Parameters	IgAV (n:44)	HC (n:34)	p-Value
Gender n (%)			
Male	20 (45.5%)	18 (61%)	0.14 *
Female	24 (54.5%)	16 (39%)	
Age (years), median (IQR)	8 (8)	7 (3)	0.22 **
Skin involvement, n (%)	44 (100%)		
Gastrointestinal involvement, n (%)	18 (40.9%)		
Joint involvement, n (%)	10 (22.7%)		
Kidney involvement, n (%)	18 (40.9%)		
Mild nephritis, n (%)	7 (15.9%)		
Moderate nephritis, n (%)	11 (25%)		
Kidney biopsy, n (%)	11 (25%)		
Membrane attack complex (ng/mL), median (IQR)	0 (219.33)	17.58 (929.97)	0.11 ^+^
Factor H (ng/mL), median (IQR)	357.31 (228.32)	543.08 (504.05)	**<0.001 ^+^**
Mannose-binding lectin (ng/mL) median (IQR)	555 (255)	626.92 (520)	0.07 ^+^
Mannose-related serine protease-1 (ng/mL), median (IQR)	65 (40.76)	62.36 (42.94)	0.24 ^+^

IgAV: immunoglobulin A vasculitis; HC: healthy controls; IQR: interquartile range. **: independent samples *t*-test. *: chi-square test. ^+^: Mann–Whitney U test.

**Table 2:** Comparison of serum complement protein levels among clinical subgroups of IgAV patients and HCs.

Parameters (ng/mL)	Moderate Nefrit (n:11)	Mild Nephritis (n:7)	IgAVwN (n:26)	HC (n:34)	p-Value
Membrane attack complex, median (IQR)	0 (0)	0 (243.58)	1.84 (301.52)	17.58(929.97)	0.08 *
Factor H, median (IQR)	334.56 (178.86)	338.49 (226.46)	430.21 (305.61)	543.08 (504.05)	0.009 *
Mannose-binding lectin, median (IQR)	537.31 (142)	527.41 (206)	574.88 (350)	626.92 (520)	0.21 *
Mannose-related serine protease-1, median (IQR)	61.87 (17.33)	60.90 (70.23)	75.74 (46.19)	62.36 (42.94)	0.48 *

IgAVwN: immunoglobulin A vasculitis without nephritis; HCs: healthy controls, IQR: interquartile range. *: Kruskal–Wallis Test. Post-hoc p values, Factor H: Dunn test with Bonferroni correction p-value of the pairwise comparison moderate nephritis vs. HC: 0.024; Dunn test with Bonferroni correction p-value of the pairwise comparison moderate nephritis vs. mild nephritis: 1.000; Dunn test with Bonferroni correction p-value of the pairwise comparison mild nephritis vs. HC: 0.388; Dunn test with Bonferroni correction p-value of the pairwise comparison mild nephritis vs. IgAVwN: 1.000; Dunn test with Bonferroni correction p-value of the pairwise comparison moderate nephritis vs. IgAVwN: 1.000; Dunn test with Bonferroni correction p-value of the pairwise comparison IgAVwN vs. HC: 0.093.

**Table 3:** Renal histopathology and serum complement protein levels in IgAVN patients undergoing biopsy.

Pathological Features	n (%)	Membrane Attack Complex (sC5b-9) (ng/mL) (Median (IQR)
Mesangial hypercellularity		
M0	7 (63.6%)	0 (0)
M1	4 (36.4%)	0 (311.86)
Endocapillary hypercellularity		
E0	5 (45.5%)	0
E1	6 (54.5%)	0 (103.95)
Segmental glomerulosclerosis		
S0	10 (90.1%)	0 (0)
S1	1 (9.1%)	-
Tubular atrophy/interstitial fibrosis		
T0	10 (90.1%)	0 (0)
T1&2	1 (9.1%)	-
Crescent		
C0	5 (45.5%)	0 (0)
C1&2	6 (54.5%)	0 (104.12)
IF		
IgA ++	4 (36.4%)	0 (0)
IgA +++	3 (27.3%)	0 (0)
IgA ++++	4 (36.4%)	0 (0)
IF		
C3 +	7 (63.6%)	0 (0)
C3 -	4 (36.4%)	0 (0)

M1, mesangial hypercellularity; E1, endocapillary hypercellularity; S, segmental glomerulosclerosis/adhesion; T, severity of tubular atrophy/interstitial fibrosis; C, presence of crescent. IQR: interquartile range. In the renal biopsy IF examination of IgA and C3 staining results, ‘-’ indicates no positivity; ‘+’ indicates mild positivity; ‘++’ indicates moderate positivity; and ‘+++’ and ‘++++’ indicate strong and very strong positivity, respectively. Statistical analysis was not conducted because of the limited sample size.

## Data Availability

All data generated or analyzed during this study are included in this published article. Additional information can be obtained from the corresponding author upon reasonable request. The data are not publicly available due to patient privacy concerns and institutional data protection policies.
